# Redefining the transcriptional regulatory dynamics of classically and alternatively activated macrophages by deepCAGE transcriptomics

**DOI:** 10.1093/nar/gkv646

**Published:** 2015-06-27

**Authors:** Sugata Roy, Sebastian Schmeier, Erik Arner, Tanvir Alam, Suraj P. Parihar, Mumin Ozturk, Ousman Tamgue, Hideya Kawaji, Michiel J. L. de Hoon, Masayoshi Itoh, Timo Lassmann, Piero Carninci, Yoshihide Hayashizaki, Alistair R. R. Forrest, Vladimir B. Bajic, Reto Guler, FANTOM Consortium, Frank Brombacher, Harukazu Suzuki

**Affiliations:** 1 Division of Genomic Technologies, RIKEN Center for Life Science Technologies, 1-7-22 Suehiro-cho, Tsurumi-ku, Yokohama 230-0045, Japan; 2 Riken Omics Science Center, 1-7-22 Suehiro-cho, Tsurumi-ku, Yokohama 230-0045, Japan; 3 Massey University, Institute of Natural and Mathematical Sciences, Auckland, New Zealand; 4 King Abdullah University of Science and Technology (KAUST), Computational Bioscience Research Center (CBRC), Computer, Electrical and Mathematical Sciences and Engineering Division (CEMSE), Thuwal, Saudi Arabia; 5 International Centre for Genetic Engineering and Biotechnology (ICGEB), Cape Town Component, Cape Town, South Africa; 6 University of Cape Town, Health Science Faculty, Institute of Infectious Diseases and Molecular Medicine (IDM), Division of Immunology, Cape Town, South Africa; 7 Riken Preventive Medicine and Diagnosis Innovation Program (PMI), 1–7–22 Suehiro-cho, Tsurumi-ku, Yokohama 230-0045, Japan

## Abstract

Classically or alternatively activated macrophages (M1 and M2, respectively) play distinct and important roles for microbiocidal activity, regulation of inflammation and tissue homeostasis. Despite this, their transcriptional regulatory dynamics are poorly understood. Using promoter-level expression profiling by non-biased deepCAGE we have studied the transcriptional dynamics of classically and alternatively activated macrophages. Transcription factor (TF) binding motif activity analysis revealed four motifs, NFKB1_REL_RELA, IRF1,2, IRF7 and TBP that are commonly activated but have distinct activity dynamics in M1 and M2 activation. We observe matching changes in the expression profiles of the corresponding TFs and show that only a restricted set of TFs change expression. There is an overall drastic and transient up-regulation in M1 and a weaker and more sustainable up-regulation in M2. Novel TFs, such as *Thap6, Maff*, (M1) and *Hivep1, Nfil3, Prdm1*, (M2) among others, were suggested to be involved in the activation processes. Additionally, 52 (M1) and 67 (M2) novel differentially expressed genes and, for the first time, several differentially expressed long non-coding RNA (lncRNA) transcriptome markers were identified. In conclusion, the finding of novel motifs, TFs and protein-coding and lncRNA genes is an important step forward to fully understand the transcriptional machinery of macrophage activation.

## INTRODUCTION

Macrophages can be phenotypically polarized by the microenvironment to activate specific functional programs that are broadly classified into two main groups, classically activated macrophages (M1) and alternatively activated macrophages (M2)(1,2). M1 is induced by IFN-gamma stimulation (M(IFNγ)), whereas M2 by IL-4 and/or IL-13 stimulation (M(IL-4), M(IL-13) and M(IL4/IL-13)) (3). M1 are characterized by the secretion of pro-inflammatory mediators and the release of killing effector function, which is associated with the control of acute infections (4). In contrast, M2 are immuno-modulators, poorly microbiocidal, can reside and proliferate in tissues, support Type2-mediated disease, homeostasis and thermogenesis (5–7). After containment of infection by M1, M2 plays a crucial role for the reduction of inflammation by following synthesizing trophic factors, increase endocytic clearance capacity, reduced pro-inflammatory cytokine secretion and also down-regulation of T cell responses (8–10). Importantly, macrophage activation towards M1 or M2 is controlled on an enzymatic level by competing for the common substrate l-Arginine by NOS2, induced by IFNγ or Arginase 1, induced by IL-4/IL-13, respectively. Considering the hostile milieu inside M1, some intracellular pathogens are able to manipulate the transcriptional network of macrophages towards an M2 fate by inducing Arginase1 in an IL-4/IL-13-independent manner to achieve persistence and subsequently development of chronic disease (11). Over the last few decades, knowledge of the transcriptional reprogramming of macrophage polarization, induced by environmental stimuli, has been accumulated by microarray-based gene expression profiling (1,12,13). Transcriptomes have contributed immensely through large consortia such as ImmGen (14) or the Human Immunology Project Consortium (15) by compiling large data sets and defining the core transcriptional program in murine macrophage and dendritic cells under steady state (16,17). Key transcription factors (TFs) and effectors involved in both stimulations have been analysed to some extent, using mice or human macrophage cells (1,18–20). The TFs IRF, NFκB, AP-1 and STAT family are known to be essential for macrophage activation (20). M1 activation leads to the induction of members of the IRF's family of TFs, such as Irf1, Irf2, Irf5, Irf7 and Irf8, which are involved in a variety of biological processes, including modulation of immune responses (19,21–23). On the other hand, M2 activation leads to the induction of Irf4 (24). Nfκb is a global activator in M1 activation, leading to the induction of Nfκb transcription factor and Nfκb pathway (25). In contrast, activation of Stat3 and Stat6 lead to the inhibition of Nfκb in M2 (26). The Stat family of TFs have a variety of biological roles in macrophage activation (20). Interferon receptor IFNAR1/2 activation by IFN leads to the activation of Stat1 in M1 and following phosphorylation Stat1 associate with CBP/P300, which binds to the promoter region of IFNγ inducible genes, recruited by histone acetylase (27,28). In contrast, IL-4/IL-13-stimulated macrophages bind to their receptor tyrosine kinases and stimulate the activation of Stat3 and Stat6 (29). The TFs Myc (30) and Tfec (31) play an important role as transcriptional regulator for M2. The TF JunB, which belongs to the AP-1 family, has been identified as a key transcriptional modulator for both classical and alternative activation (32). Others, like Hif1A is present in inflammation and metabolism networks of M1 (33). Despite a large number of studies on macrophage activation, in reference to classical or alternative activation, a transcriptional model for macrophage activation has not yet been achieved, mainly due to limited time course studies. Hence, a more systematic analysis to understand the dynamics of transcriptional regulation in classical and alternative macrophages is required.

Recently the FANTOM5 consortium mapped transcription start sites of 975 human and 399 mouse samples to generate a comprehensive promoter expression atlas which provides expression profiles for known, novel, coding and non-coding transcripts (34). It also identified active enhancer elements among these cell types (35). Classical, intermediate and non-classical monocytes were used to examine the landscape of coding, non-coding and transcribed enhancers in these populations (36). In those transcriptome analyses, CAGE (capped analysis of gene expression) technology, with the method for non-amplified CAGE library construction, was subjected to the single molecule Helicos sequencer (non-biased deepCAGE). Here, as a satellite study within the FANTOM5 phase 2 activity, which defined the dynamics of enhancer and promoter activity during mammalian cellular activation and differentiation (37), we focused on the analysis of transcriptional regulation and marker genes, as well as transcribed long non-coding RNAs (lncRNAs) during classical and alternative activation in murine primary macrophages. DeepCAGE analysis allowed us to identify regulatory motifs and distinct sets of TFs in M1 and M2, which may regulate their transcriptional machinery. Promoter-based gene expression analysis allowed us to identify new transcription marker genes and lncRNA genes in IFNγ- and IL-4/IL-13-stimulated macrophages. Taken together our CAGE transcriptome analysis reconceived our current understanding of macrophage activation. The work is part of Functional Annotation of Mammalian Genome (FANTOM5) project. Data, genomic tools, and co-published manuscripts are summarized online at http://fantom.gsc.riken.jp/5/.

## METERIALS AND METHODS

### Generation of bone marrow-derived macrophages (BMDMs)

BALB/c mice were purchased from Jackson Laboratories and bred in South Africa. Mice were sacrificed in accordance with the Animal Research Ethics of South African National Standard (SANS 10386:2008) and University of Cape Town of practice for laboratory animal procedures. The protocol (Permit Number: 012/036) was approved by the Animal Ethics Committee, Faculty of Health Sciences, University of Cape Town, Cape Town, South Africa. Bone marrow-derived macrophages were generated from 8–12 week old BALB/c male mice as described previously (38). Briefly, bone marrow cells were harvested from femurs. Cells were cultured for 10 days at 37 ^o^C under 5% CO_2_ in PLUTZNIK differentiation media (DMEM containing 10% FCS, 5% horse serum, 2 mM L-glutamine, 1 mM Na-pyruvate, 0.1 mM 2-betamercaptoethanol, 30% L929 cell-conditioned medium, 100 U/ml penicillin G, 100 μg/ml streptomycin) in 140 mm x 20 mm petridishes with vent (Nunc, Denmark). After 10 days, BMDMs were harvested and plated in 6-well tissue culture plates (Nunc, Denmark). Each well was seeded with 2 × 10^6^ BMDMs for subsequent stimulation.

### BMDMs stimulation with IFNγ or IL-4/IL-13

The harvested BMDMs were plated in 6-well plates for overnight incubation. Following incubation cells were either left untreated or stimulated with IFNγ (100 unit/ml, BD Biosciences, San Jose, CA, USA) or IL-4/IL-13 (100 units/ml each, BD Biosciences, San Jose, CA), IL-4 (100 units/ml, BD Biosciences, San Jose, CA, USA), IL-13 (100 units/ml, BD Biosciences, San Jose, CA, USA) and incubated at 37°C under 5% CO_2._ At 0, 2, 4, 6, 12, and 24 hours post stimulation, BMDMs were lyzed with 700 μl of Qiazol (Qiagen, Valencia, CA, USA) and stored at minus 80°C for RNA extraction. Total RNA was prepared using miRNAeasy kit (Qiagen, Valencia, CA, USA) and its concentration and quality was measured using nanodrop and bioanalyser, respectively. Total RNA was used for CAGE library preparation.

### Preparation of Helicos CAGE library and sequencing

CAGE libraries for single molecule sequencing were prepared, sequenced, mapped and clustered into TSS regions as described previously (37). Briefly, in this study, libraries were prepared by manual and automated protocols using 5 μg of total RNA, with RIN value of more than 7.5 (Supplementary Table S1A). Sequencing was carried out using the HeliScope Single Molecule Sequencer platform. Three to four biological replicates were used per time point. Reads corresponding to ribosomal RNA were removed using the rRNAdust program. Remaining CAGE reads were mapped to the genome (mm9) using Delve (http://fantom.gsc.riken.jp/software/). Reads mapping with a quality of less than 20 (<99% chance of a true match) were discarded. Furthermore, all reads that mapped to the genome with a sequence identity of <85% were discarded.

### Construction of promoter data

To identify peaks (TSS clusters) in the CAGE profiles, we used decomposition peak identification (DPI) as described previously in the time-course paper (37). This method identifies local regions producing signals continuously along the genome and estimates a limited number of CAGE profiles which underline all observed biological states by independent component analysis, and determining peaks based on the estimated profiles.

The ‘relative log expression (RLE)’ method (39) to calculate normalization factors for the expression of promoters was used in this study. This method calculates a relative expression score to the geometric mean of all samples yielding a scaling factor for each sample that is used to adjust the median value in each sample. During the normalization procedure in the current study, the same methodology was employed but with calculation of geometric mean taken from the previous FANTOM5 phase 1 study (34), in order to make it possible to compare normalized expression in this study with the samples from FANTOM5 phase 1. The same strategy was used in our recently published analysis of the FANTOM5 phase 2 samples (37).

### Principal component analysis

Principal component analysis (PCA) was performed using the R-package ‘psych’. Each number in the figure represents average expression (triplicate) of each sample in one time point. Each stimulation has a different color. The components shown are rotated using the ‘varimax’ rotation. The dispersion ellipses are calculated (R-package ‘vegan’) using the standard deviation of point scores and the correlation defines the direction of the principal axis of the ellipse.

### Motif activity analysis

Motif activities were calculated as described previously (40,41). Briefly, we assume transcription factors (TFs) regulate the expression of promoters through binding to DNA sequence elements in proximal regions. The expression of a promoter in a sample is assumed to be a linear function of the number of conserved TF binding sites in the proximity of the promoter. Specifically, we assume that

\begin{equation*} E_{p,s} = noise + c_p + c_s + \sum\nolimits_m {(N_{p,m} *A_{m,s} )} \end{equation*}

where *e_p,s_* is the logarithm of the expression of each promoter *p* in sample *s*, the noise is assumed to be normally distributed with the same standard deviation for all features in the sample, *c_p_* is a promoter dependent constant, *c_s_* is a sample dependent constant, and *N_p,m_* is the predicted number of functional binding sites for motif *m* that appear in promoter *p*. The expression level was determined by CAGE, and the motif activities of known motifs are fitted to the data using all promoters that are significantly expressed in at least one of the samples. The motif activities represent sample-dependent abilities of motifs to regulate expression levels. Using the inferred activities and their standard deviations, for each motif a *z*-score is calculated representing the contribution of each motif to expression changes across the time course.

### Differential expression analysis of TFs and Non-TFs protein-coding marker genes

Differential expression (DE) analysis was performed after discarding all promoters that do not have at least 5 tags mapped to them in at least one library. These promoters were not deemed reliable or of interest. For each gene we pooled the expression of its associated promoters by summing their tags to create one tag count for each gene. Promoters not associated to genes were discarded. In each individual comparison we only considered genes for differential expression analysis, if the sum of tags of all libraries in the respective comparison was more than 10 tags (42). This filtered out lowly expressed promoters in the conditions that get compared to make the analysis more robust (42). Gene expression analysis was performed using the Bioconductor package edgeR (39) (www.bioconductor.org). We compared each time point of IFNγ- and IL-4/IL-13-stimulated BMDM (2, 4, 6, 12 and 24 h) with non-stimulated BMDMs at 0 h to obtain DE genes of TF and non-TF candidates. A log_2_ fold-change > 1 (log_2_ fold < -1 in case of down-regulation) and false discovery rate (FDR) < 0.05 were used as thresholds to define differentially expressed TF up- and down-regulated in IFNγ- and IL-4/IL-13-stimulation based on the edgeR calculations. Differential expressed up- and down-regulated non-TF genes in IFNγ and IL-4/IL-13 stimulation were obtained using a log_2_ fold-change > 2 (log_2_ fold <-2 in case of down regulated) and a FDR of < 0.05.

### Differential expression analysis of lncRNA promoters

Mouse lncRNAs from GENCODE release M2 (http://www.gencodegenes.org/mouse_releases/2.html) was used for analysis. To convert genome positions of mouse genome assembly mm10 to mouse genome assembly mm9, we applied the UCSC LiftOver tool (http://hgdownload.cse.ucsc.edu/admin/exe/linux.x86_64/liftOver). Then, the CAGE tags were mapped to the lncRNA transcript set. A typical CAGE tag was considered as associated with a gene if it intersects with the region that covers [–500,+500] bp around transcription start site (TSS) of transcript on the same strand. When one TSS is associated with multiple CAGE clusters, we associate only one CAGE cluster based on the nearest distance between TSS and 5′ end of the CAGE cluster. The CAGE expression of a given TSS is defined as the sum of the CAGE tags associated with the CAGE cluster. To identify the differentially expressed lncRNA genes, we compared the IFNγ- and IL-4/IL-13-stimulated BMDMs (at 2, 4, 6, 12 and 24 h) against non-stimulated macrophage control at 0 h to obtain significantly up- or down-regulated promoters of lncRNA transcripts. We retained only those transcripts that had non-zero expression level in at least two replicas of any of the compared groups. We discard transcripts having low expression values while keeping only those that had at least 1 tag per million (TPM) reads in at least two samples of the considered group. The gene expression is normalized using the Trimmed Mean of *M*-values (TMM) method. Statistical analysis of gene expression data to identify DE genes was performed using the edgeR (39) R package. EdgeR's extract Test method was used to evaluate differential expression, while the resulting *P*-values were adjusted for multiple comparisons testing using the Benjamini-Hochberg FDR < 0.05.

## RESULTS

### Construction of promoter activity profiles for classically and alternatively activated macrophages

**Figure 1.**
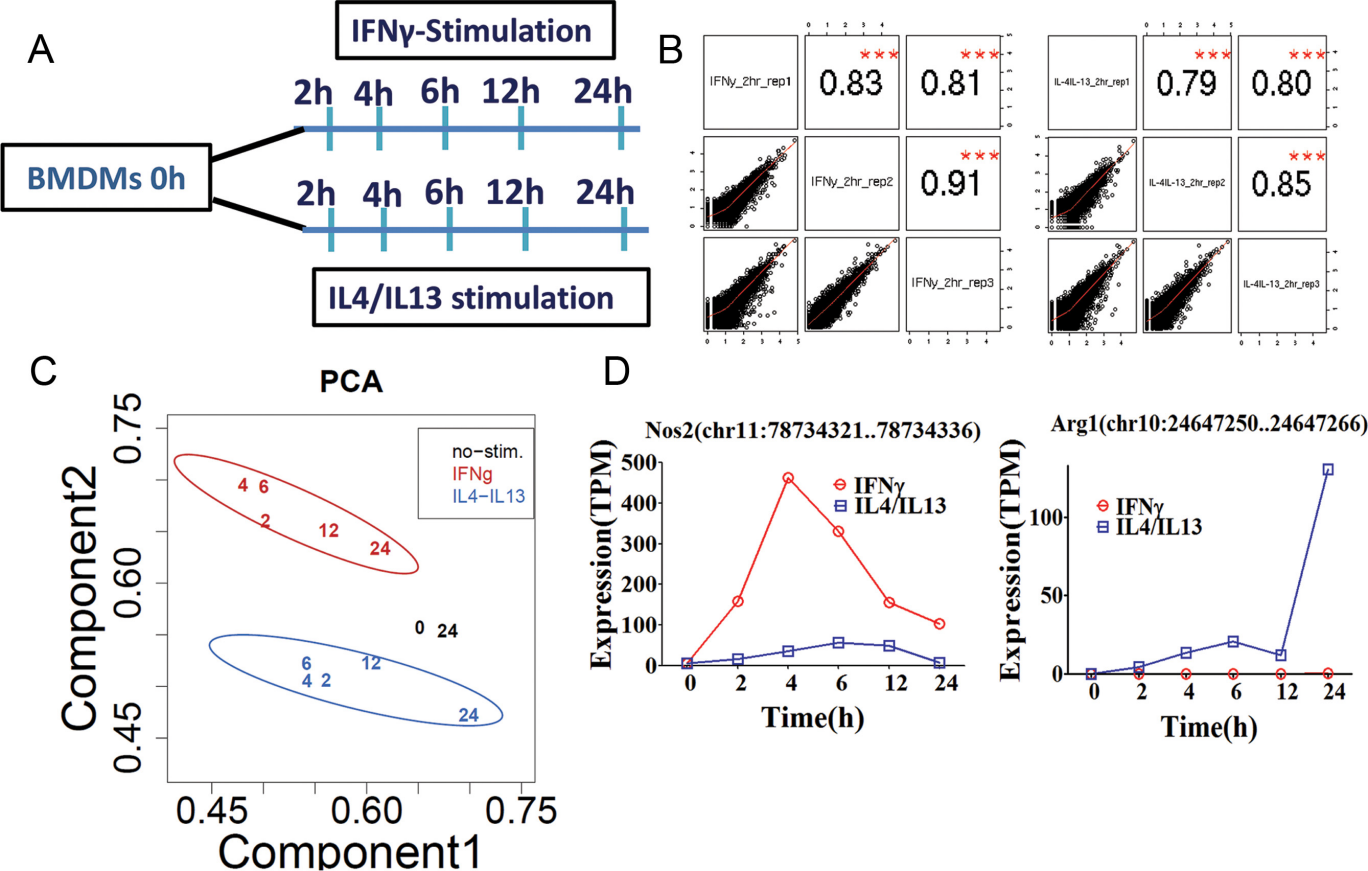
Experimental design and quality control. (**A**) Schematic representation of the preparation and stimulation of BMDMs from BALB/c mice. After 10 days of differentiation, BMDMs were stimulated with IFNγ or IL-4/IL-13. At 2, 4, 6, 12 and 24 h post-stimulation, total RNA was collected followed by non-amplified deepCAGE analysis. Zero hour non-stimulated BMDMs were used as control. Three independent biological experiments were analyzed to obtain the promoter activity. (**B**) Biological replicates were plotted to obtain the relative Pearson correlation coefficients among the replicates. (**C**) Principal component analysis (PCA) was performed using IFNγ-stimulated macrophages (M1), IL-4/IL-13-stimulated macrophages (M2) and non-stimulated macrophages and the separation of M1, M2 and non-stimulation based on component 1 and component 2 is shown (see Material and Methods). Each number in the plot represents the average expression (triplicates) of each sample in one time point. Each of the stimulations has a different color. (**D**) Promoter expression profiles of classical activation marker gene *Nos2*, and alternative activation marker gene *Arg1*, are shown. The expression profiles of promoters are represented by tags per million (TPM). The data was obtained from three biological experiments and was plotted as mean expression.

To understand the transcriptional regulation of classical and alternative activation, mouse bone marrow-derived macrophage cells (BMDMs) were harvested after stimulation with IFNγ (100 units/ml for M1) or IL-4/IL-13 (100 units/ml for M2) in a time-dependent manner. The time course samples (0, 2, 4, 6, 12 and 24 h) were subjected to the non-amplified deepCAGE measurement using single molecule Helicos sequencers (Figure 1A). For this analysis we only consider those libraries that had at least 500 000 uniquely mapping tags (ranged from 713 918 to 16 279 576 with a median of 1 927 283 tags; Supplementary Table S1A). The mapped CAGE tags were computationally clustered to establish promoter activity profiles (see methods). The data was reproducible among three biological replicates with satisfactory correlations (Pearson correlation coefficient > 0.71 to 0.95) (Figure 1B and Supplementary Figure S1). Principal component analysis (PCA) demonstrated that IFNγ-stimulated M1 (M(IFNγ)) clearly separated from the IL-4/IL-13-stimulated M2 (M(IL-4/IL-13)) (Figure 1C and Supplementary Figure S2A). Unstimulated 0 and 24 h samples were very closely mapped to each other in the PCA plot, indicating that time-dependent shift of the PCA plot in M(IFNγ) and M(IL-4/IL-13) may not be cell cultured-dependent changes. This is supported by DE analysis between unstimulated 0 and 24 h control, which revealed only a negligible number (only 6) of promoters to be altered (Supplementary Table S1B). Next, we explored promoter level expression profiles for well-known marker genes. As expected, promoter expression profiles for typical M1 marker genes, such as *Nos2, Tnf, Cxcl9, Cxcl10* and *Cxcl11* (Figure 1D and Supplementary Figure S3), and M2 marker genes, such as *Myc, Mrc1, Arg1, Ccl22* and *Ccl24* (Figure 1D and Supplementary Figure S3), were drastically up-regulated by IFNγ- and IL-4/IL-13-stimulation, respectively, confirming cytokine-induced macrophage activation and polarization. Finally, although we have also taken IL-4 only and IL-13 only data for M2, the PCA plot revealed that IL-4-, IL-13- and IL-4/IL-13-stimulated M2 clustered together (Supplementary Figure S2B), indicating that IL-4 and IL-13 had mainly overlapping gene expression profiles. Based on the finding, we used M(IL-4/IL-13) as representative of M2 for further analysis.

### Identification of important motif activities involved in M(IFNγ) and M(IL-4/IL-13)

To understand transcriptional regulation involved in M(IFNγ) and M(IL-4/IL-13), the promoter expression profiles were subjected to Motif Activity Response Analysis (MARA). Briefly, assuming that TFs regulate expression of transcripts through binding to DNA sequence motifs, expression is modeled as a linear function of the number of predicted DNA binding sites in their proximity, yielding an activity profile across all samples for each DNA binding motif interrogated (details in Material and Methods). Because the activity dynamics of motif and expression profile of TF(s) regulating the motif are considered to be similar, this makes it possible to determine which TFs are the most active in regulating the expression dynamics of the system. Motif activity changes also depend upon other deterministic factors, such as localization and modification of the associated TFs. The overall contribution of each motif within each event is calculated as *z*-scores (40,41), i.e. the average number of standard deviations of the motif activity from the zero mean across the time course.

Motif activity and *z*-score values were calculated independently in the M(IFNγ) and M(IL-4/IL-13) (Supplementary Tables S2A and S2B, respectively), and listed top motifs of *z*-score > 3 in each profile (Table 1 for M(IFNγ) and Table 2 for M(IL4/IL13)). In order to focus on the active motifs involved in IFNγ- or IL-4/IL-13-stimulation, motifs with higher activity change were selected by calculating delta motif activity by subtracting minimum motif activity value from maximum motif activity value. This resulted in five motifs with delta motif activity change > 0.15 in either stimulation, NFKB1_REL_RELA, IRF1,2, IRF7, TBP and FOS_FOS{B,L1}_JUN{B,D} (marked by yellow in Tables 1 and 2). Interestingly, four out of five motifs, except for FOS_FOS{B,L1}_JUN{B,D}, were selected in both M(IFNγ) and M(IL-4/IL-13), suggesting that the same motifs could play an important role even in different macrophage polarizations. Further, three out of five motifs, NFKB1_REL_RELA, IRF1,2 and IRF7 were involved in the top 10 active motifs of non-stimulated BMDM (Supplementary Figure S4), which were derived from the FANTOM5 phase 1 motif activity analysis in comparison with other cell types and tissues (34), indicating that limited number of active motifs in non-stimulated BMDM are also involved in macrophage polarization.

**Table 1.**
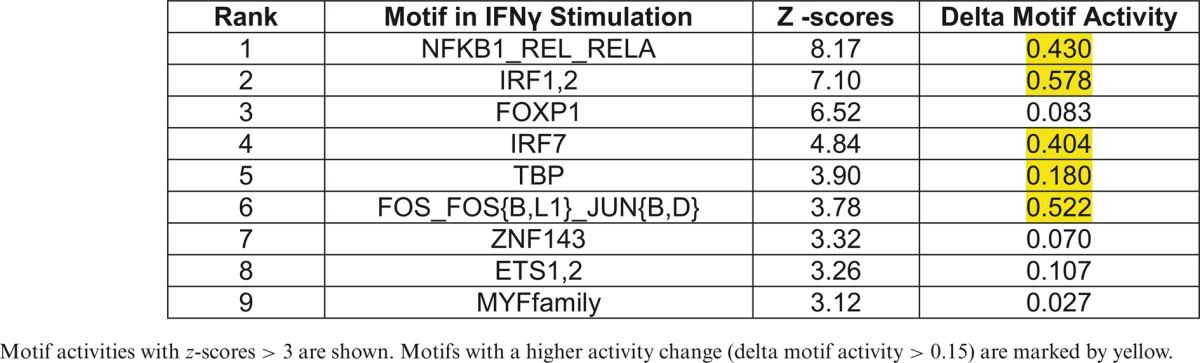
The top motif activities in classical macrophage activation

Motif activities with *z*-scores > 3 are shown. Motifs with a higher activity change (delta motif activity > 0.15) are marked by yellow.

**Table 2.**
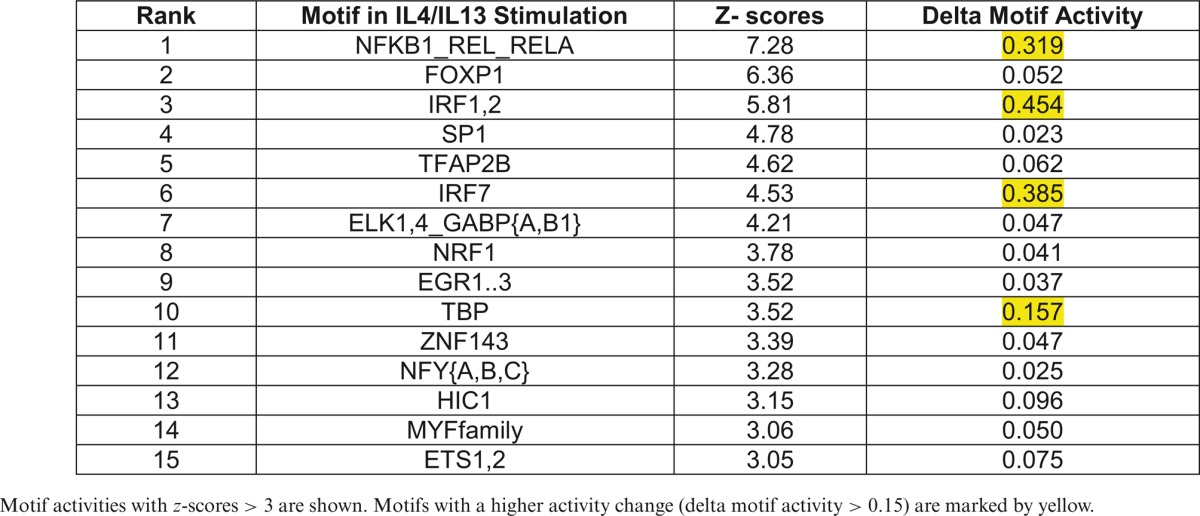
The top motif activities in alternative macrophage activation

Motif activities with *z*-scores > 3 are shown. Motifs with a higher activity change (delta motif activity > 0.15) are marked by yellow.

**Figure 2.**
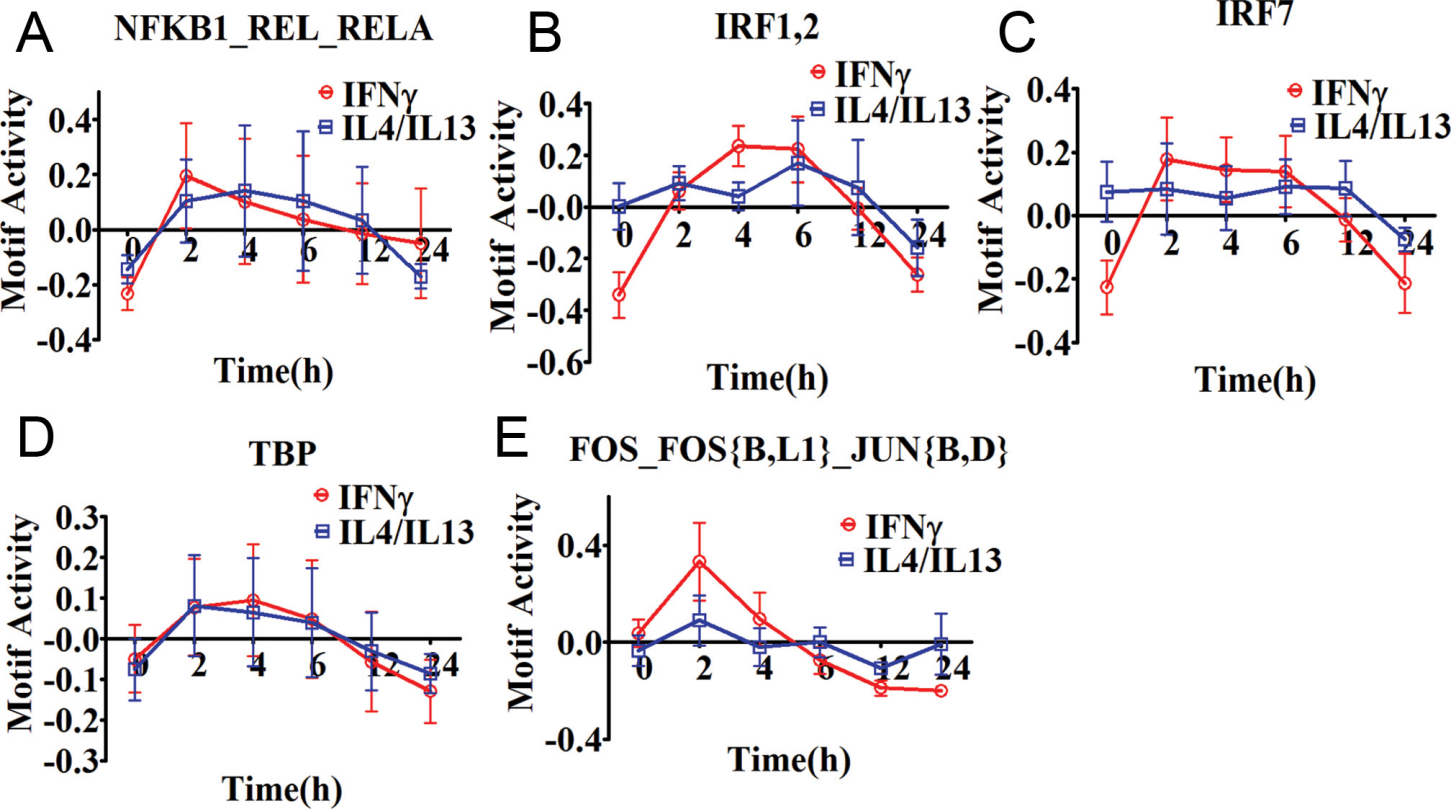
Motif activity response analysis of M(IFNγ) and M(IL-4/IL-13). Motif activity response analysis was performed using promoter activity profiles of M(IFNγ) and M(IL-4/IL-13), obtained from CAGE data. The identified top 5 motif activities with high activity change (z-acore >3 and delta motif activity change > 0.15) are shown in (**A**) NFKB1_REL_RELA, (**B**) IRF1,2, (**C**) IRF7, (**D**) TBP and (**E**) FOS_FOS{B,L1}_JUN{B,D}. The data is obtained from three independent biological experiments and plotted as mean ± SEM. The motif activity is calculated as relative value at each time point where summation of values for each stimulation series becomes zero.

Of interest, all selected five motifs of M(IFNγ) (red lines in Figure 2) presented a common drastic increase in their activity within 2 h of stimulation. Thereafter, the dynamics changed depending on the motif. Two motifs, NFKB1_REL_RELA (Figure 2A) and FOS_FOS{B,L1}_JUN{B,D} (Figure 2E), slowly decreased their motif activity. The three remaining motifs, IRF1,2, IRF7 and TBP (Figure 2B, C and D) kept their high motif activity between 2 to 6 h during IFNγ-stimulation, and decreased thereafter drastically. The dynamics for the motifs of M(IL-4/IL-13) (blue lines in Figure 2) had no common motif dynamics but NFKB1_REL_RELA and TBP were similar to M(IFNγ), with a drastic increase in their activity within 2 h of stimulation followed by a decline. In contrast, the motifs, IRF7, IRF1,2 and FOS_FOS{B,L1}_JUN{B,D} revealed weak motif activity increases during IL-4/IL-13-stimulation, with small changes between 2 and 12 h. Thus, most of these motifs seem to be more commonly used, with distinct motif activity changes within different macrophage polarizations.

### Expression analysis of TFs associated with motifs from MARA analysis

**Figure 3.**
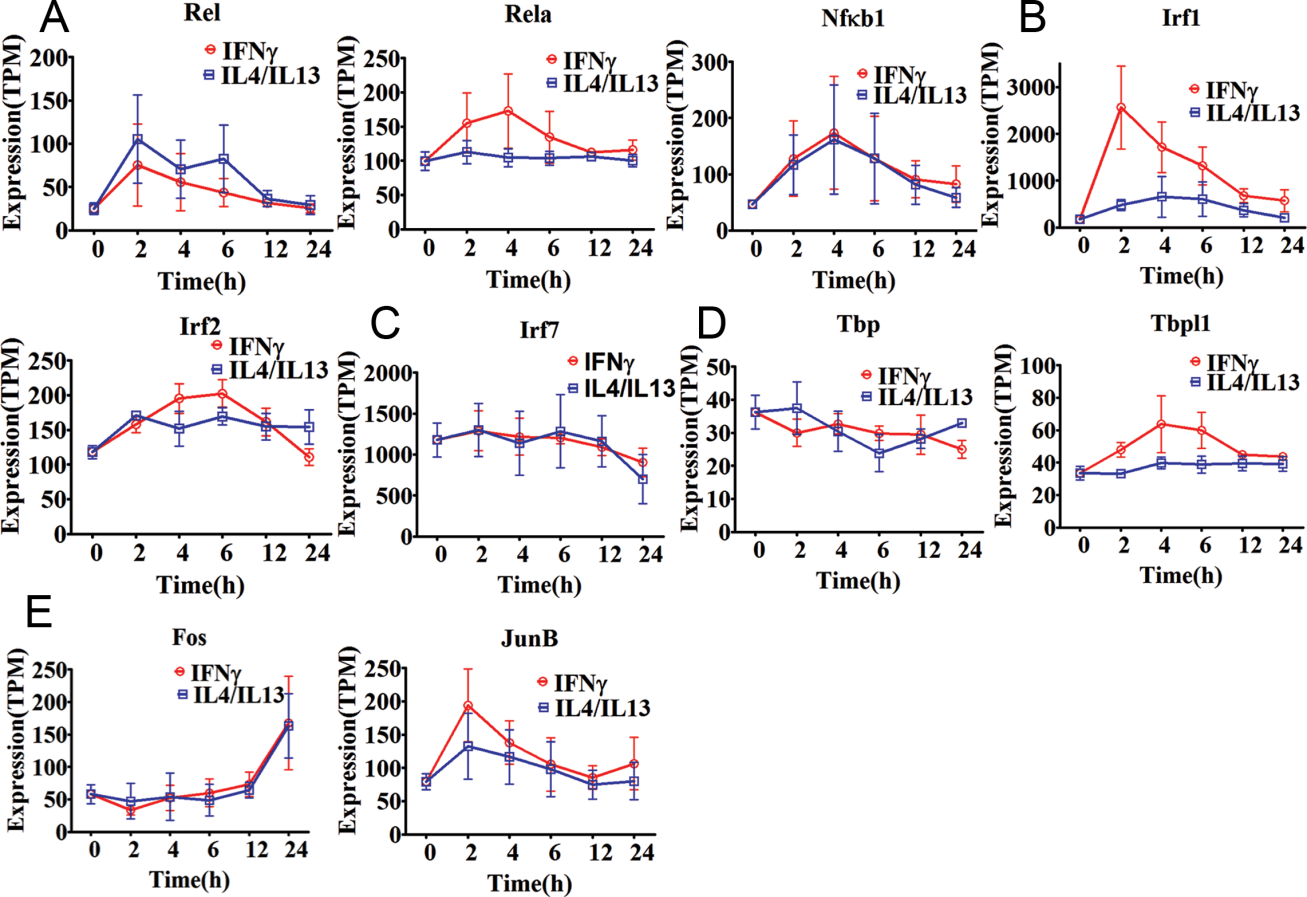
Expression profiles of transcription factors associated with top five motif activities. Expression of the associated transcription factor genes is shown as tags per million (TPM). Error bars were calculated based on the standard error of three replicates. (**A**) Transcription factors *Rel, Rela* and *Nfκb1* associated with NFKB1_REL_RELA motif activity. (**B**) Transcription factors *Irf1* and *Irf*2 associated with IRF1,2 motif activity. (**C**) Transcription factor *Irf7* associated with IRF7 motif activity. (**D**) *Tbp* and *Tbpl1* associated with TBP motif activity. (**E**) *Fos, Fosl1, FosB, JunB* and *JunD* associated FOS_FOS{B,L1}_JUN{B,D} motif activity. *Fos* and *JunB* are shown whereas *FosB*,*Fosl1* and *JunD* are not shown because the expression remains close to the detection limit throughout the time course in M(IFNγ) and M(IL-4/IL-13).

Each motif activity is mediated by a concentration of active/workable TFs, associated with the motif, where expression level of the TFs is one of the important contributing determinants. To identify TFs responsible for the observed motif activity change, gene expression profiles of TFs associated with the five motif activities were explored (Figure 3).

The three TFs, *Nfκb1, Rel and Rela* are associated with the NFKB1_REL_RELA motif and initially up-regulated with a subsequent down-regulation in M(IFNγ) (red lines in Figure 3A), as expected from the motif activity. Of interest, expression dynamics of *Nfκb1* was indistinguishable between M(IFNγ) and M(IL-4/IL-13). *Rel* and *Nfκb1* revealed similar expression changes to that of M(IFNγ) and *Rela* showed relatively constant expression in M(IL-4/IL-13) (blue lines in Figure 3A). Together, these results suggest that distinct TFs, Rel/Rela/Nfκb1 and Rel/Nfκb1, may be involved in the motif activity change in M(IFNγ) and M(IL-4/IL-13), respectively (Figure 2A). Sustained high expression of *Rel*, from 2 to 6 h of stimulation in M(IL-4/IL-13), was particularly consistent with the motif activity change. Moreover, the two TFs, Irf1 and Irf2, associated with IRF1,2 motif and the expression dynamics of both *Irf1* and *Irf2* in M(IFNγ) indicated a cooperative responsibility for the drastic change seen in the IRF1,2 motif activity (red lines in Figures 2B and 3B). Furthermore, relatively mild up-regulation of both TFs was consistent with weak changes in the motif activity of M(IL-4/IL-13) (blue lines in Figures 2B and 3B), This may indicate that IRF1,2 motif activity changes in M(IFNγ) and M(IL-4/IL-13) are due to expression changes of both TFs. We also found that the FOS_FOS{B,L1}_JUN{B,D} motif activity change may be dominantly regulated by the associated TFs. Expression of the associated TFs, *Fos* and *JunB* was present (Figures 2E and 3E), whereas for example *FosB, Fosl1* and *JunD* expression remained close to the detection limit throughout the time course in M(IFNγ) and M(IL-4/IL-13). *Fos* and *JunB* showed quite different expression profiles with the *JunB* profile being similar to the motif activity profile for M(IFNγ), suggesting that JunB is mainly responsible for change of the motif activity. This confirmed a recently reported network analysis, revealing that *JunB* is required for the expression of genes involved in classical activation (32). Nonetheless, this does not exclude importance of Fos since Fos/JunB hetero-dimer is necessary for the motif activity.

The IRF7 and TBP motifs were difficult to interpret with the corresponding mRNA expression changes of the associated TFs. Almost constant mRNA expression of the associated TF *Irf7* (Figure 3C) did not match with the drastic increase and decrease of the IRF7 motif activity in M(IFNγ) (red line in Figure 2C), but matched with the M(IL-4/IL-13) (blue line in Figure 2C). On the other hand, the TBP motif activity was reflected in M(IFNγ) by mRNA expression profile of one of TBP motif associated TFs, *Tbpl1* (red lines in Figures 2D and 3D), but the motif activity changes in M(IL-4/IL-13) did not match to the mRNA expression of the associated TFs. Taken together, these results predict that distinct TFs are involved in NFKB1_REL_RELA motif activity changes in M(IFNγ) and M(IL-4/IL-13) and particularly, the NFKB1_REL_RELA, IRF1,2 and FOS_FOS{B,L1}_JUN{B,D} motif activity changes could be well explained by the associated TF expression. However, IRF7 and TBP motif activity changes were not corresponding to the respective TF expression, which may indicate that other deterministic factors, such as localization, modification, co-factors of the associated TFs and involvement of lncRNA genes as regulatory components (43), may play important roles in IRF7 and TBP regulation of stimulation response (44).

### Transcription factor expression in M(IFNγ) and M(IL-4/IL-13)

**Figure 4.**
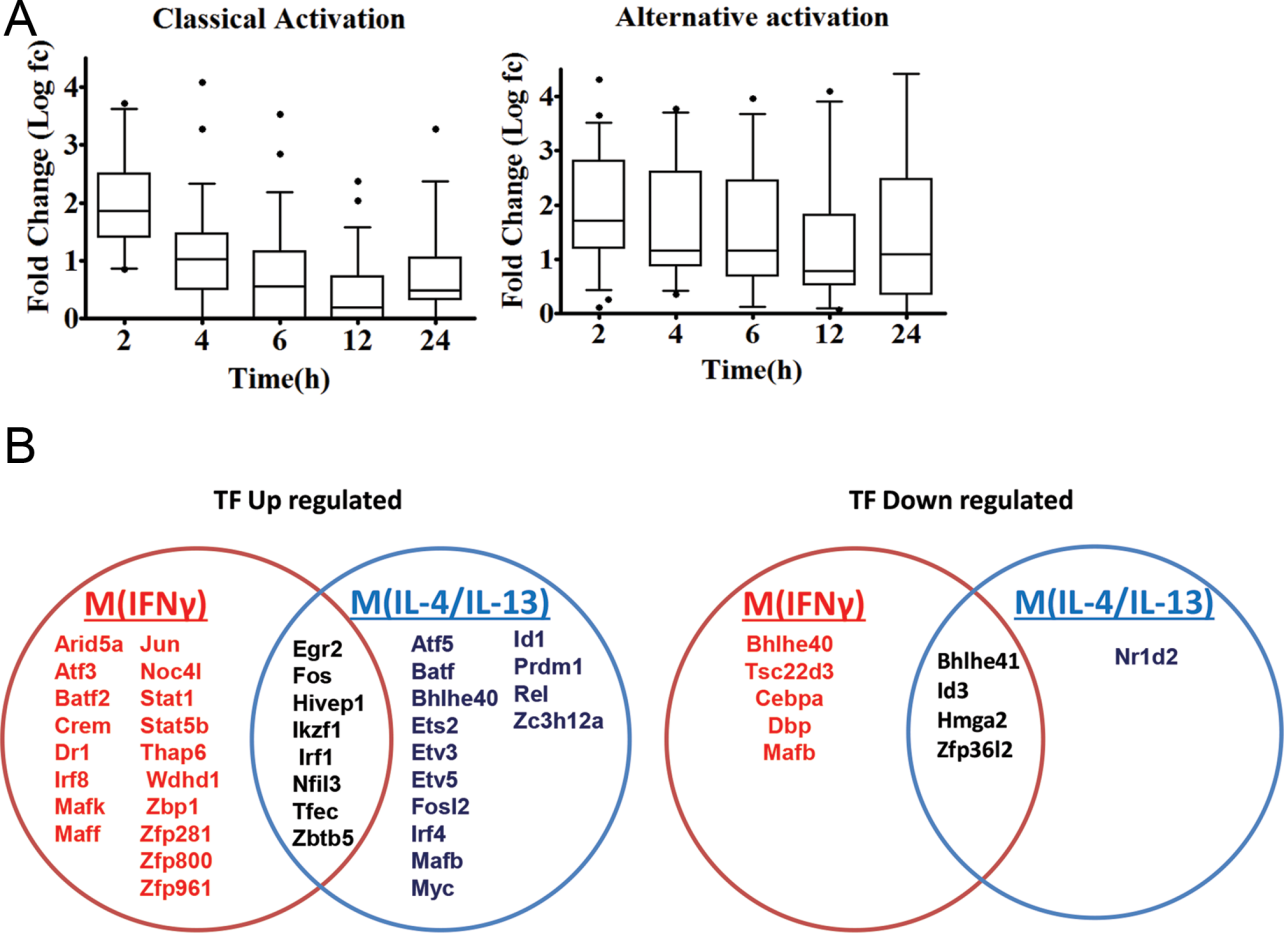
Transcription factors involved in M(IFNγ) and M(IL-4/IL-13). (**A**) Box plot analysis of the expression log fold-changes of all differentially up-regulated transcription factors in classically and alternatively activated macrophages over time (left and right panels, respectively). Boxes show median and interquartile ranges and whiskers show the 10th and 90th percentile values. (**B**) The Venn diagram shows that M(IFNγ) and M(IL-4/IL-13) up-regulate 26 and 22 (left) and down-regulate 9 and 6 (right) distinct transcription factor genes.

Although motif activity analysis is a powerful tool for insights of transcriptional regulation in classical and alternative activation, this analysis does not cover all TFs, as many TFs’ binding motifs are currently not known. To better understand the transcriptional regulation of M(IFNγ) and M(IL-4/IL-13), promoter-based gene-level TF expression were analyzed globally. All dynamic data points of M(IFNγ) and M(IL-4/IL-13) were compared with non-stimulated macrophage controls (zero hour), hence this allowed the identification of significantly up- or down-regulated TF genes. This analysis resulted in the identification of 35 and 27 TF genes, that were significantly differentially expressed (at least a 2-fold change in expression, FDR < 0.05) in M(IFNγ) and M(IL-4/IL-13), respectively (Tables 3 and 4 and Supplementary Table S3A and S3B). Most of the TFs revealed up-regulation in both polarization (26/35 = 74.3% for M(IFNγ) and 22/27 = 81.5% for M(IL-4/IL-13)). Considering that 3,361 promoters for 953 TF genes were expressed in BMDMs at time 0 h, the results showed that only a restricted number of TF genes change on a gene expression for both polarization events. Figure 4A shows the average expression features of up-regulated TF genes in time for M(IFNγ) and M(IL-4/IL-13). A rapid up-regulation at 2 h was evident in both macrophage polarization. However, up-regulated TF expression quickly declined thereafter in M(IFNγ), whereas more sustainable expression was characteristic for M(IL-4/IL-13) (Figure 4A). We do not know the biological importance but these differences might be the consequences of different functions between classically versus alternatively activated macrophages.

**Table 3.**
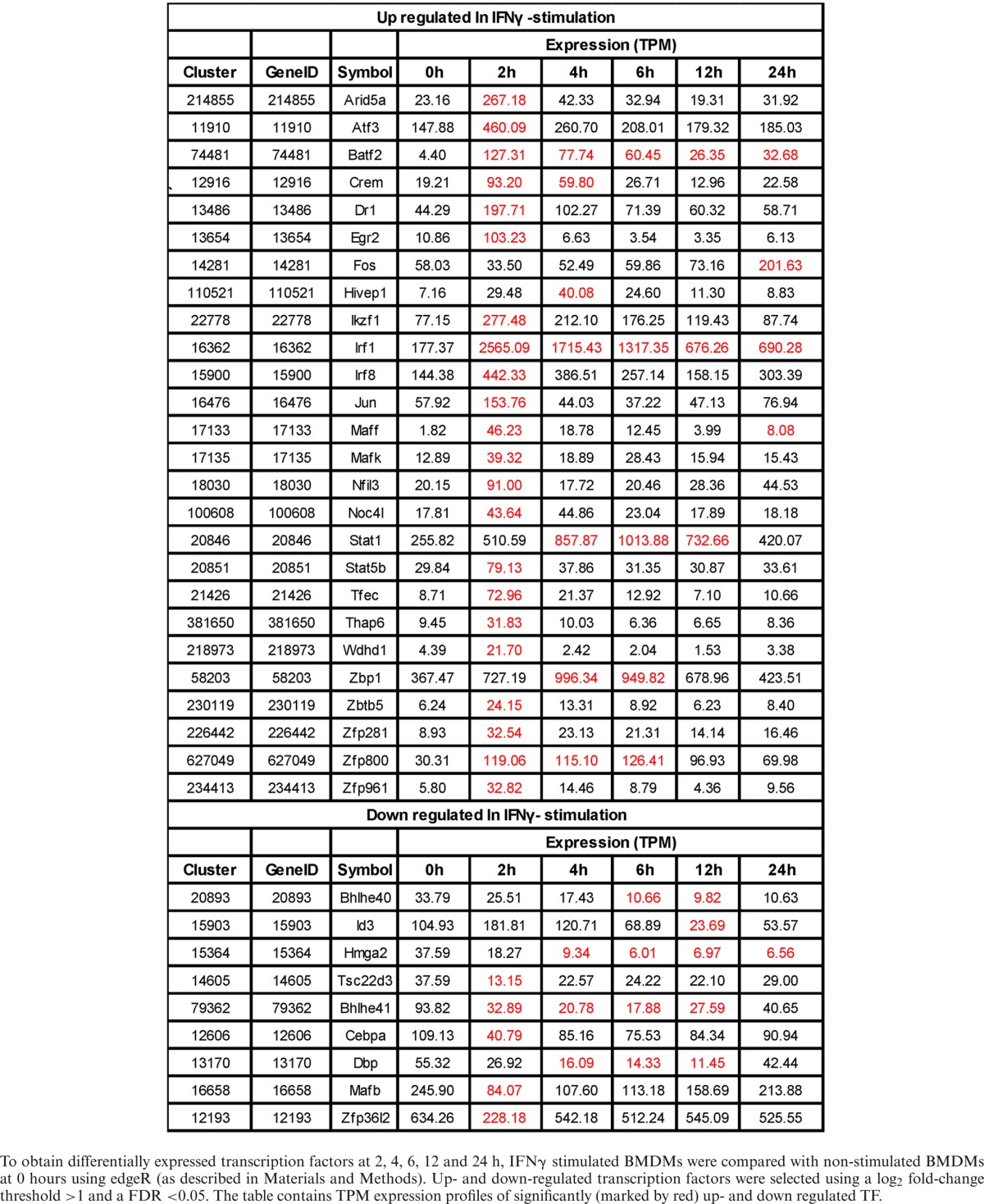
Differentially expressed transcription factor in M(IFNγ)

To obtain differentially expressed transcription factors at 2, 4, 6, 12 and 24 h, IFNγ stimulated BMDMs were compared with non-stimulated BMDMs at 0 hours using edgeR (as described in Materials and Methods). Up- and down-regulated transcription factors were selected using a log_2_ fold-change threshold >1 and a FDR <0.05. The table contains TPM expression profiles of significantly (marked by red) up- and down regulated TF.

**Table 4.**
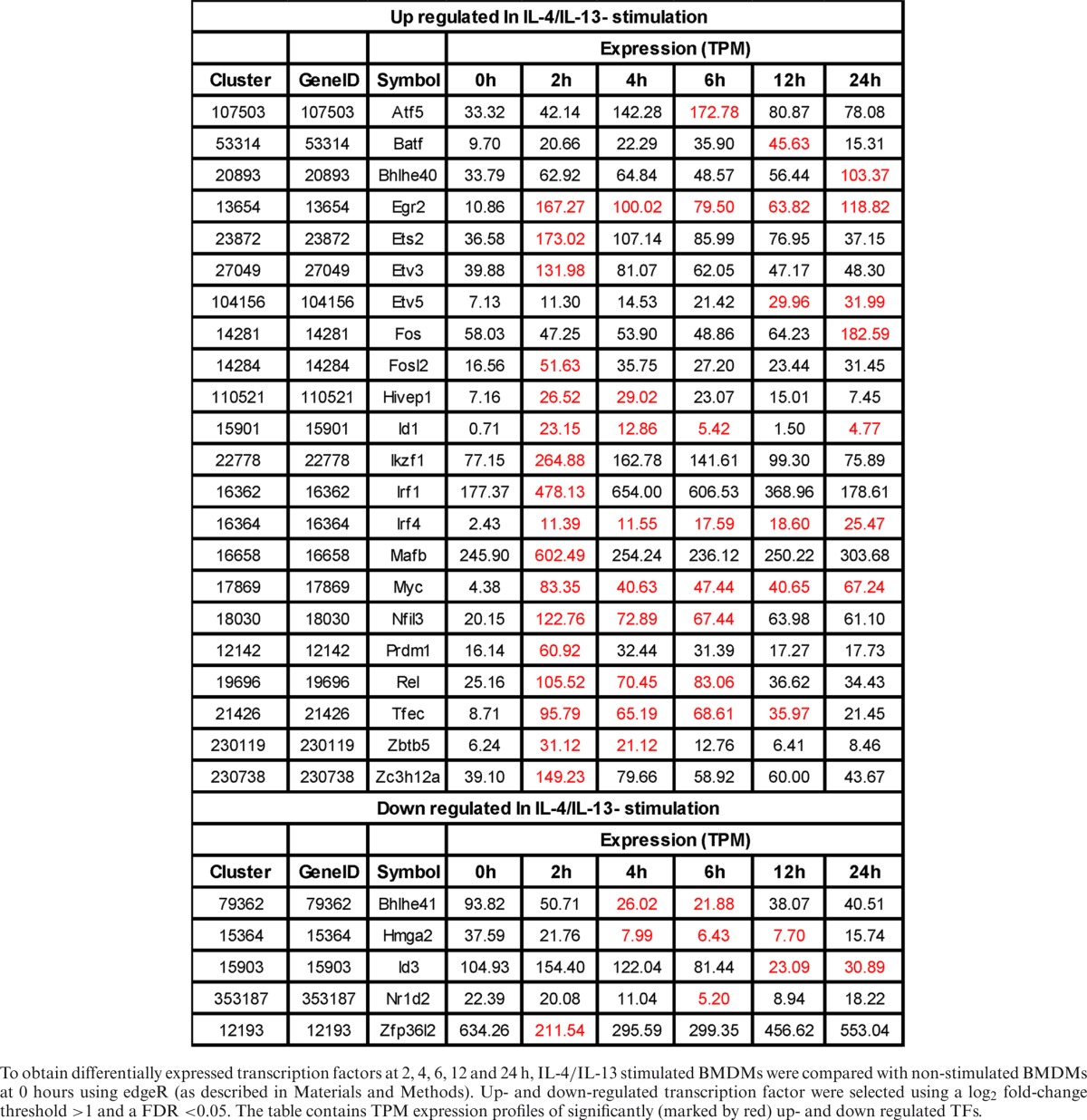
Differentially expressed transcription factors in M(IL-4/IL-13)

To obtain differentially expressed transcription factors at 2, 4, 6, 12 and 24 h, IL-4/IL-13 stimulated BMDMs were compared with non-stimulated BMDMs at 0 hours using edgeR (as described in Materials and Methods). Up- and down-regulated transcription factor were selected using a log_2_ fold-change threshold >1 and a FDR <0.05. The table contains TPM expression profiles of significantly (marked by red) up- and down regulated TFs.

Interestingly, eight TF genes were shared between M(IFNγ) and M(IL-4/IL-13) (Figure 4B), whereas the majority were distinct from each other macrophage polarization state. In addition to a few common immediate early response TF genes like *Egr2, Fos, Irf1* and *Maff* etc, there were few common TFs as transcriptional repressor genes like *Hivep1, Nfil3* and *Zbtb5* for up-regulation and *Bhlhe41* and *Id3* for down-regulation. Together, this may indicate that both polarization events need to alternate the resting state of BMDM transcriptional regulation.

Specifically up-regulated TF genes in M(IFNγ) and M(IL-4/IL-13) (Figure 4B and Tables 3 and 4) were further analyzed. TFs known to be involved in macrophage activations, such as *Stat1, Stat5a, Irf1, Irf8, Crem* and *Jun* etc. for M(IFNγ) and *Myc, Irf4, Tefec, Ets2, Etv3* and *Etv5* etc for M(IL-4/IL-13) were found. Of importance, novel TFs for M(IFNγ), such as *Thap6, Maff*, etc and novel TFs for M(IL-4/IL-13), *Hivep1, Nfil3, Rel, Batf, Bhlhe40, Prdm1* etc. were uncovered. We speculate that these TFs could be involved in specific transcriptional regulation processes for polarization events. Also of interest, several TF genes corresponding to different member of TF families were involved in either polarization. Those were Batf2, Atf3, Irf8 and Zfp800/Zfp281/Zfp961 for M(IFNγ), and Batf, Atf5, Irf4, and Zc3h12a for M(IL-4/IL-13). Together, this analysis may indicate distinct transcriptional regulatory networks of M(IFNγ) and M(IL-4/IL-13), consisting of distinct or overlapping sets of TF family proteins.

### Novel transcription marker candidates for M(IFNγ) and M(IL-4/IL-13)

The comprehensive transcriptome data was systematically analyzed to identify novel M(IFNγ) and M(IL-4/IL-13) marker transcripts, to possibly expand previous M(IFNγ) and M(IL-4/IL-13) marker sets (1,12,13,45). In order to uncover more drastic differences, the stringency was increased to >4 fold difference for at least one time point and with a FDR < 0.05 using three biological replicates (see Methods). With these criteria, 118 and 110 genes were found up-regulated in M(IFNγ) and M(IL-4/IL-13), respectively. Interestingly, most of the up-regulated genes increased expression within the first 2 h, whereas down-regulated genes were not clustered in a time-dependent manner (Supplementary Tables S4A and S4B). Similar as already observed during the motif activity and TFs, and likely as a consequence, the identified genes in M(IFNγ) were rapidly and transiently up-regulated, whereas in M(IL-4/IL-13) up-regulation was more sustainable over the time period (Supplementary Figures S5A and S5B), which might be involved in the regulation of these genes. The list includes 66 well-established classical marker genes, such as *Ifi205, Il15ra, Il27, Irg1, Tnfsf10, Tnfsf9, Tnf, Nos2, Gbp5, Stx11, Gbp7, Mmp13, Cxcl10, Ccr1* and *Ccl12* etc. and 42 well-established alternative marker genes, such as *Socs2, Cxcl2, Ccl7, Hnrpll, St7, Igf1, IL-4i1, Ccl17, Arg1, Mgl2* and *Mmp12* etc. In addition, 52 and 67 up-regulated non-TF protein coding genes were identified as novel classical and alternative transcription marker candidates, respectively. Of particular interest, the newly discovered marker candidates on a transcriptional level involved many chemokine genes, such as *Ccl4, Cxcl1, Cxcl2, Cxcl3, Ccl2, Ccl22* and *Ccl7* particular in M(IL-4/IL-13).

To understand whether the rapid and transiently up-regulated genes in M(IFNγ), both TF and non –TF genes, reflect a general inflammatory response in macrophages, they were compared with the clusters 1, 2, and 3 of rapid lipid A-induced genes in a BMDM time course study (0 to 120 min) by Bhatt *et al*. (46). Lipid A is the active component of lipopolysaccharides, known to promote inflammatory responses in macrophages. Among 139 induced genes (including TF and non-TF genes) in M(IFNγ), 91 genes (64.4%) were overlapped with rapid genes (up to 2 h) from lipid A stimulation. Gene ontology analysis for the common fraction of genes revealed significant enrichment of the ontology term, ‘immune response’ (GO:0006955), ‘inflammatory response’ (GO:0006954), ‘response to wounding’ (GO:0009611), which reflects a general inflammatory response of macrophages (Supplementary Table S5A). Interestingly, gene ontology analysis of 48 genes, induced specifically by IFNγ, revealed significant enrichment of the ontology term antigen processing and presentation via MHC class II (Supplementary Table S5B), which indeed is a very specific function for macrophages. Together, these observation suggest that the sharp response in M(IFNγ) is a more general inflammatory response of macrophages induced by IFNγ and lipid A with an additive effect of M(IFNγ) specific responses.

Of note, we also identified nine and eight lncRNAs as novel classical and alternative marker candidates, respectively (see methods). Although the function of these lncRNAs are not known, most listed lncRNAs were found significantly expressed in monocytes/macrophages and most of them specifically expressed in distinct cell types. lncRNAs ENSMUST00000181286.1 and ENSMUST00000180613.1 were identified to be highly expressed on macrophages (Supplementary Figure S6). ENSMUST00000180613.1 was found in most tissues but ENSMUST00000181286.1 specifically expressed in axillary lymph node, ileum, peyer's patch and thymus, among few others (Supplementary Figure S7). The dynamics of lncRNA response generally showed rapid (2 h) and transient responses in M(IFNγ) but slower and more sustained responses in M(IL-4/IL-13) (Supplementary Figures S5C and S5D). We also investigated the expression profile for nearby protein-coding genes for differentially expressed lncRNAs (Supplementary Figure S8 and Supplementary Table S6). We could not find overlaps with lncRNAs and protein-coding genes in most cases. In three occasions, IncRNAs ENSMUST00000180613.1, ENSMUST00000154810.1 and ENSMUST00000181286.1 overlapped with the protein coding gene NM_021524, NM_001290506 and NM_008371, respectively (Supplementary Table S6). The expression pattern of these protein coding genes did not show any clear correlation of expression with the nearest IncRNAs. Potentially, these IncRNA species may play important roles during macrophage polarization events and can be included as transcriptional markers for classical and alternative activation due to their significant differential expression.

## DISCUSSION

In a time course transcriptomic approach using CAGE, we compared the dynamics of IFNγ-activated classical macrophages and IL-4/IL-13-activated alternative macrophages. Motif Activity Response Analysis (MARA), which was already used within a pervious FANTOM study, identified important TF binding motifs involved in transcriptional regulation of monoblast-monocyte differentiation (40) and allowed the identification of five motifs, NFKB1_REL_RELA, IRF1,2, IRF7, TBP and FOS_FOS{B,L1}_JUN{B,D} and their corresponding transcription factors. They seemed to play important roles during transcriptional regulation of macrophage polarization, as three of them, namely Nfkb1, Irf1 and Irf7, were previously implicated to be regulators of classical activation (18–23). The TBP motif associates with TATA-binding proteins (TBP), core of TFIID and part of the RNA Polymerase II pre-initiation complex, hence important for gene expression (47). We analysed other time course projects in FANTOM5 for the TBP motif to explore whether the observed big activity change may be specific to macrophage activation. The motif activity change was also observed in three out of nine other time course projects, which was T cells differentiation, in vitro differentiation of embryonic stem cells to neuron and tracheal to ciliated epithelium activation. This may indicate that high TBP motif activity change is not a general event, but may prone to the few specific time courses, including macrophage activation.

Interestingly, we found in this time course study that four out of five mentioned motifs were indeed involved not only during classical but also during alternative activation. Thus, most of these motifs seem to be more commonly used during polarization, but with distinct activity dynamics. This may be an efficient way to regulate different polarization events using restricted number of TFs, which however, influences many genes involved in classical and alternative macrophage activation. Besides the five motifs we uncovered, we also identified other highly significant and reproducible (*z* > 3) motifs with polarization specificity. Those were SP1, TFAP2B, ELK1,4,_GABP{A,B1}, NRF1, EGR1..3, NFY{A,B,C} and HIC1 motifs specific for alternative activation (Table 2). Although the activity changes were relatively small, these motifs may play significant role in the transcriptional regulation of alternative activation. An example, we found that Egr2, the associated TF with EGR1..3 motif, showed significant up-regulation specifically in alternative activation (Table 4).

Differences in the motif activity dynamics were partly explained by expression changes of the associated TF genes. However, our results also suggest that localization, modification and co-factors of the associated TFs may work as important deterministic factors (40,44). The transcription factor Batf2 was highly expressed in M(IFNγ) and a clear marker gene as not expressed in M(IL-4/IL-13). Batf2 is known to compensate for Batf3 in CD8^+^ and CD103^+^ dendritic cell development during *T. gondii* infection (48). Interestingly, in this study, TFs Batf2 and Irf1 were both specifically up-regulated in M(IFNγ), and we demonstrated by co-immuno-precipitation that the Batf2 associates with Irf1 and positively regulates downstream genes crucial for classical activation (49). Murphy *et al*. showed that physical association between Batf and Irf4 plays an important role in transcriptional regulation for T-cell differentiation, where Batf3 can compensate the role of Batf (48). Together with our findings that different members of TF families are specifically up-regulated in either classical or alternative activation, TFs Batf and Irf4, both specifically up-regulated in M(IL-4/IL-13) may also cooperatively regulate downstream genes involved in alternative activation. The concept of combinatorial regulation, now well accepted, may explain our findings that up-regulated downstream genes can be quite specific in both activations, with similar important motifs involved. Although some of the specifically up-regulated TFs may regulate downstream genes through unidentified distinct motifs, exploration of cooperation for these TFs with the identified important motifs may pave the way for further understanding the complex transcriptional regulatory mechanisms in both activations.

Motif activity analysis does not cover all TFs, as many TFs’ binding motifs are currently not known. In this regard, we identified gene expression of 26 and 22 TFs, which were significantly up-regulated in M(IFNγ) and M(IL-4/IL-13). Among them, many have been reported to play functional roles in macrophage biology. For example, Irf4 was expressed in macrophages following M(IL-4/IL-13) stimulation, which supports previous findings of its involvement in priming to an alternative macrophage phenotype (50,51). Of note, Myc was strongly induced in M(IL-4/IL-13) with high tag per million (TPM) reads, which supports a previous study showing that Myc expression is required for alternative polarization of macrophages (30). Others, like transcription factors Nfil3, and Zc3h12a, an RNase, which were also highly expressed in M(IL-4/IL-13), could possibly be involved in the down-regulation of Th1 responses by transcriptionally inhibiting IL-12p40 in macrophages (52–55). The transcription factor Tfec was previously found to be induced by IL-4 and IL-13 or LPS in BMDM (31). This is in line with our finding; however Tfec was also induced following IFNγ- and IL-4/IL-13-stimulation. TF Arid5a was induced in macrophages in response to LPS, IL-1β, and IL-6. Arid5a was strongly induced following IFNγ-stimulation and able to promote inflammatory responses through the induction of IL-6 in macrophages (56). Rel has previously been shown to be induced during classical macrophage polarization, controlling the induction of Tnf (57). In stimulated Rel^−/−^ peritoneal effusion macrophages also regulates IL-6 and TNF-alpha expression but GM-CSF, G-CSF, nitric oxide, production and cytotoxic activity remain normal. We confirmed in this work that Rel is an important transcription factor in both M1 and M2. In addition, we found well-known TFs regulating macrophage polarization such as Stat1 that were robustly expressed in classically activated macrophages (58) and Irf8 shown to regulate macrophage inflammatory response (59). Among the differentially expressed transcription factors, Irf1, Irf8, Batf2, Arid5a, Stat1 and Atf3 in M(IFNγ) (Table 3) and Egr2, Irf1, Mafb, Myc and Ets2 in M(IL-4/IL-13) (Table 4) were highly expressed indicating that these TFs may have central role in regulating transcription network of M1 and M2, respectively. Taken together, these differentially expressed TFs must be involved in transcriptional regulation of M1 and M2.

Due to our time course promoter-based comprehensive transcriptome analysis, we systematically identified transcripts, which were crucially involved in classical and alternative activations. In addition to the significantly up-regulated novel non-TF protein-coding genes, we successfully identified for the first time several lncRNAs that showed activation specific up-regulation at similar level as those of protein-coding genes. Because most of lncRNAs are believed to be involved in feedback transcriptional regulation (43), functional perturbation analysis of these newly identified lncRNAs will enable us for a better understanding of the role of these transcripts in macrophage activation, to gain a more comprehensive understanding of transcription regulation mechanism for both activations. Moreover, these differentially expressed lncRNAs can serve as transcription markers of each of these macrophage activations.

The novel CAGE-based transcriptomics approach, together with comprehensive bioinformatics techniques, such as MARA, allowed for a deeper understanding of transcriptional regulation in these polarization events, and extended our current comprehension of these processes. In summary, we identified important TF motifs for regulation of the transient activation; inferred potentially responsible TFs associated with the motifs; uncovered novel TFs that appeared specific to each activation event, and expanded on specific transcription marker genes, including lncRNAs for both polarizations. The promoter-based comprehensive transcriptome data of macrophage activations will be a valuable resource for the research community, particular in immunology.

## Supplementary Material

SUPPLEMENTARY DATA
