# Supplementary material for: Redefining the transcriptional regulatory dynamics of classically and alternatively activated macrophages by deepCAGE transcriptomics
Source: Nucleic Acids Res. 2015 Jun 27;43(14):6969–82. doi: 10.1093/nar/gkv646 (PMC4538831; doi:10.1093/nar/gkv646)
Supplement: SUPPLEMENTARY DATA [file supp_43_14_6969__index.html]

Redefining the transcriptional regulatory dynamics of classically and alternatively activated macrophages by deepCAGE transcriptomics — SUPPLEMENTARY DATA 

# Redefining the transcriptional regulatory dynamics of classically and alternatively activated macrophages by deepCAGE transcriptomics

## SUPPLEMENTARY DATA

- SUPPLEMENTARY DATA
- SUPPLEMENTARY DATA
- SUPPLEMENTARY DATA
- SUPPLEMENTARY DATA
- SUPPLEMENTARY DATA
- SUPPLEMENTARY DATA
