# Supplementary material for: Redefining the transcriptional regulatory dynamics of classically and alternatively activated macrophages by deepCAGE transcriptomics
Source: Nucleic Acids Res. 2015 Jun 27;43(14):6969–82. doi: 10.1093/nar/gkv646 (PMC4538831; doi:10.1093/nar/gkv646)
Supplement: SUPPLEMENTARY DATA [file supp_gkv646_nar-00633-h-2015-File006.pdf]

Supplementary Information

Redefining the transcriptional regulatory dynamics of classically and alternatively activated macrophages by deepCAGE transcriptomics.

Supplementary Figure S1

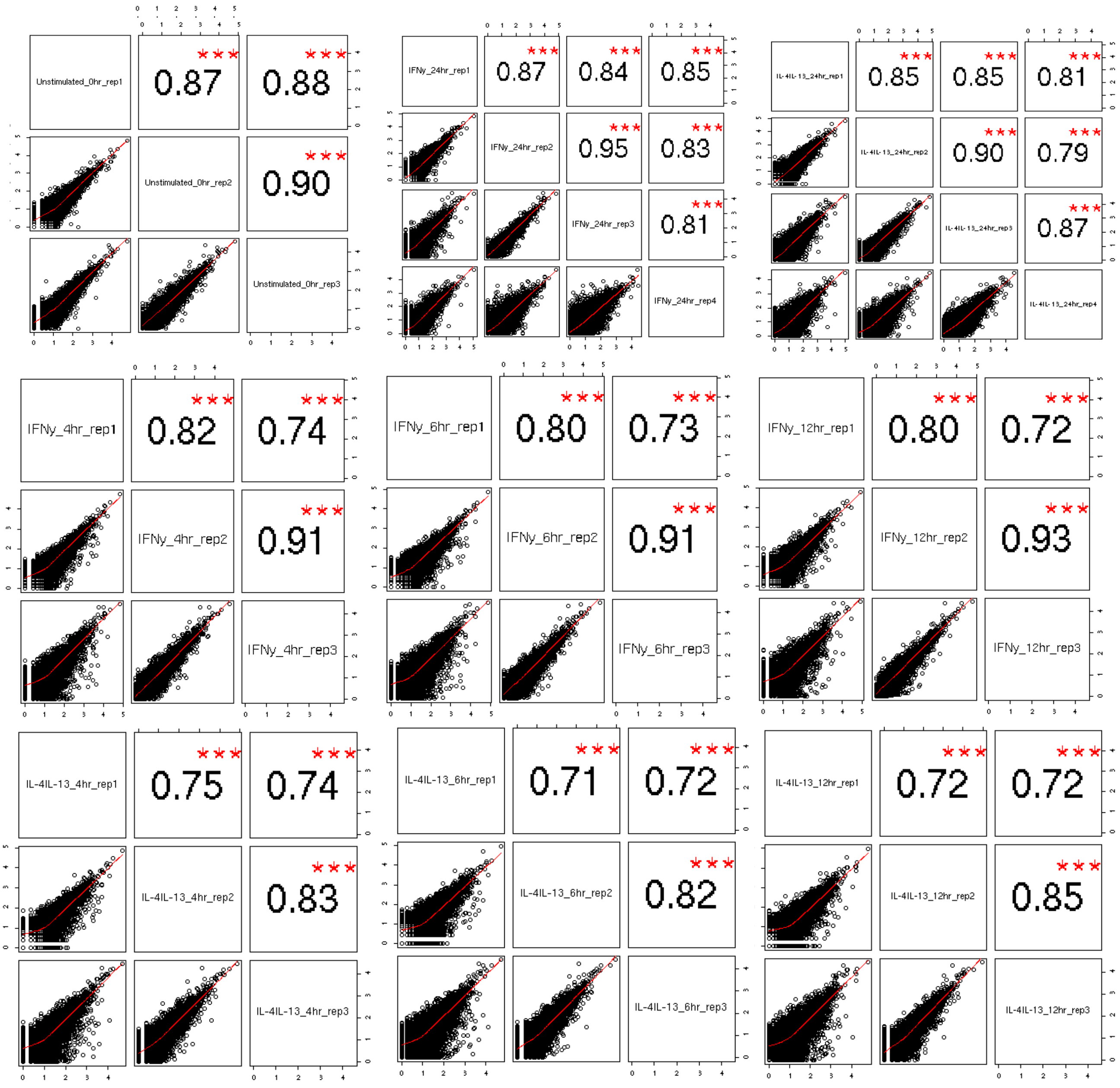

Supplementary Figure S1 CAGE data correlation among replicates. Biological replicates of CAGE data were plotted at each time point to get the relative Pearson correlation.

Supplementary Figure S2

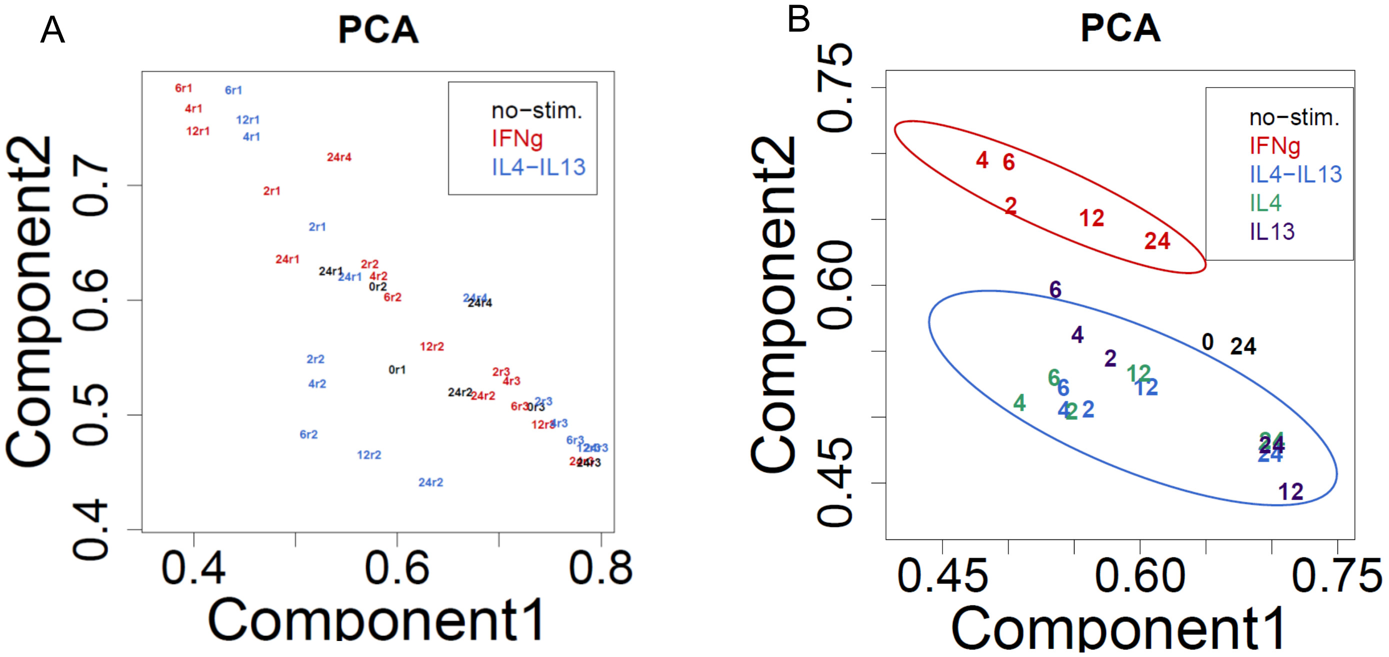

Supplementary Figure S2. Principal component analysis (PCA). **(A)** Three replicates data, indicated as r1, r2 or r3, is shown for M(IFNγ) and M(IL-4/IL-13). **(B)** The PCA plot for IFNγ-stimulation (M1) and IL4-, IL13- or IL-4/IL-13-stimulation (M2). Each number in the plot represents the average expression (triplicate) of each sample in one time point.

Supplementary Figure S3

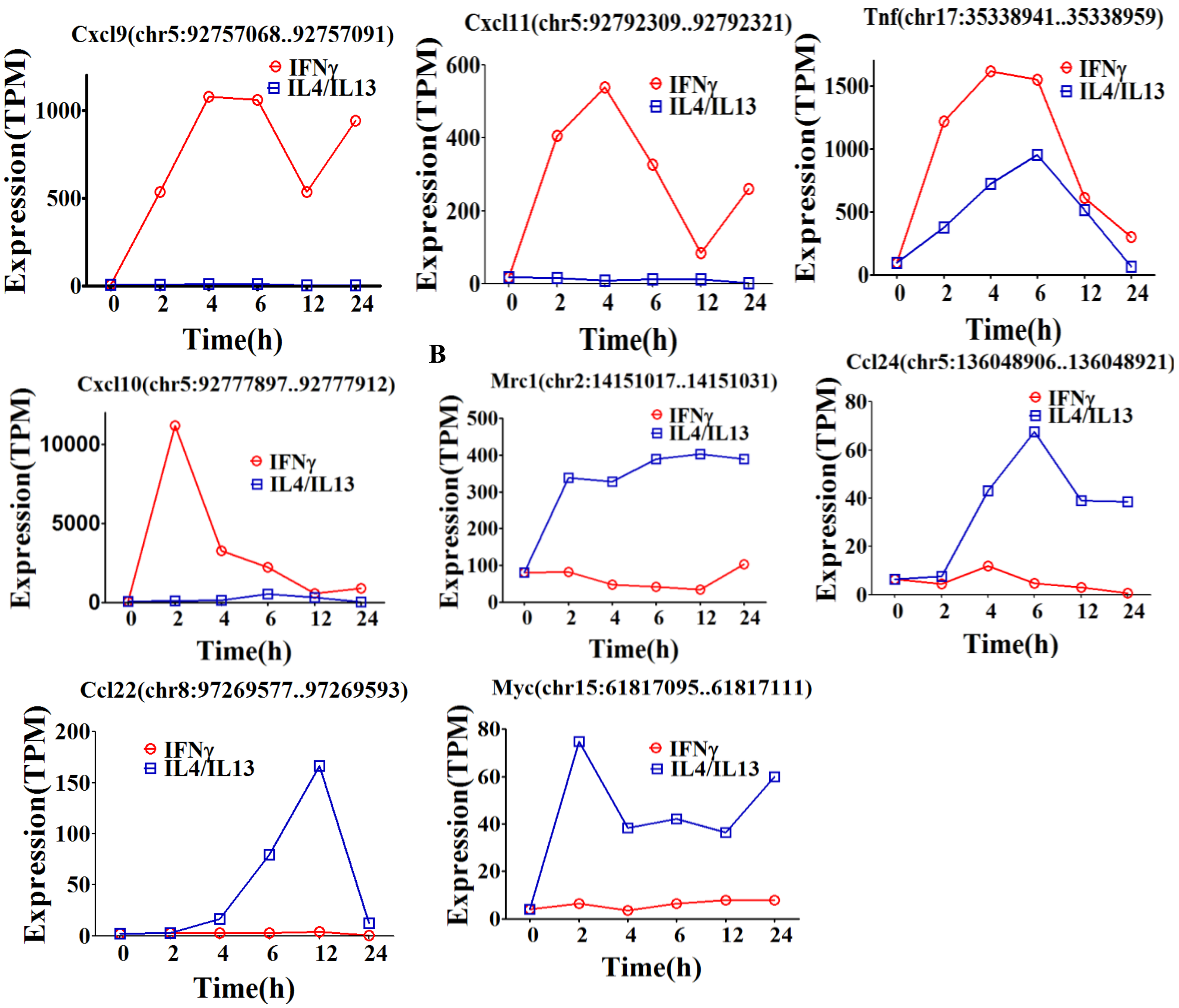

Supplementary Figure S3. Markers of classical and alternative activation. Promoter expression profiles of typical classical marker gene *Cxcl9, Cxcl11, Tnf*, *Cxcl10* and alternative marker gene *Mrc1*, *Ccl24, Ccl22*, *Myc* are shown. Expression level of promoters is represented by Tags per Million (TPM). The data obtained from three biological experiments was plotted as mean expression.

Supplementary Figure S4

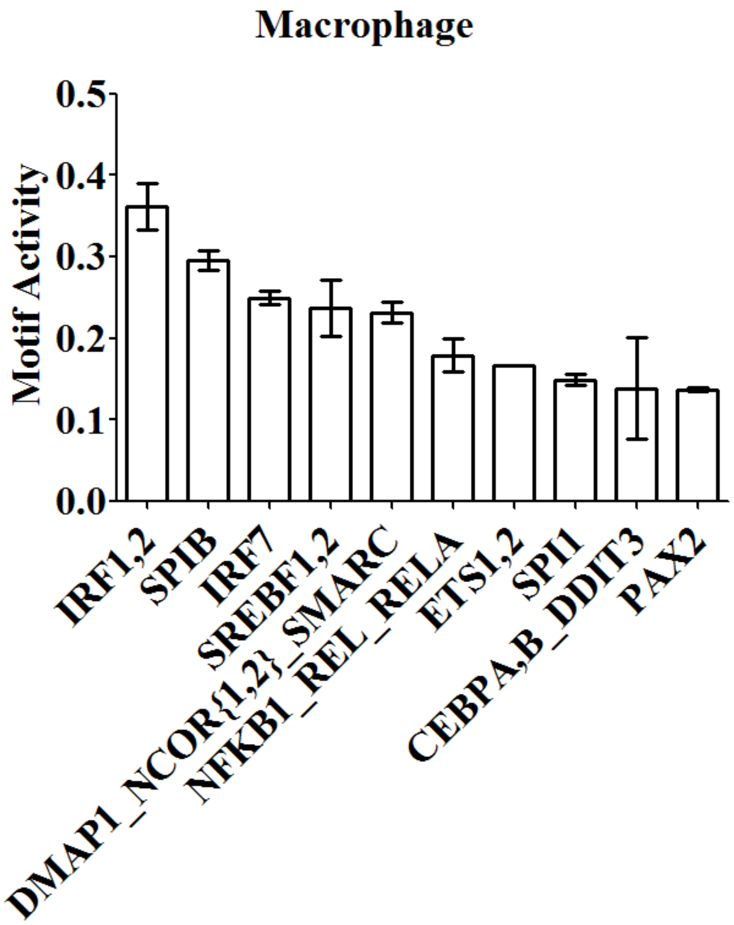

Supplementary Figure S4. Top 10 active motif activities of non-stimulated BMDM, derived from the FANTOM5 phase 1 MARA analysis.

Supplementary Figure S5

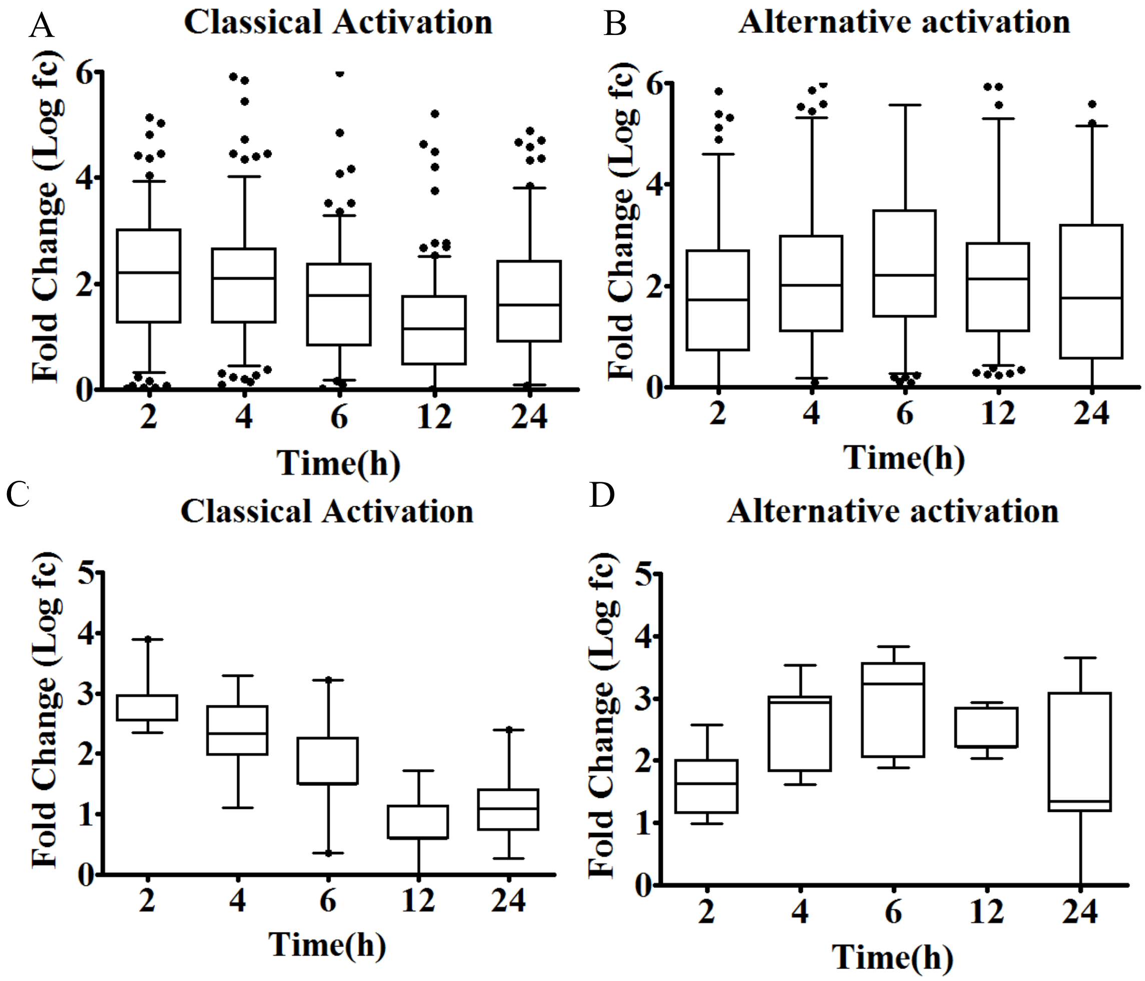

Supplementary Figures S5. Box plot analysis of differentially up-regulated non-TF and lncRNA markers in classical and alternative activation. Boxes show median and interquartile range and whiskers show the 10 th and 90 th percentile values. (A) and (C) Differentially up-regulated non TF and lncRNA in classically activated macrophages, respectively (shown in supplementary Tables S4A and S6, respectively). (B) and (D) Differentially up-regulated non-TF and lncRNA in alternatively activated macrophages, respectively (shown in supplementary Tables S4B and S6, respectively).

Supplementary Figure S6

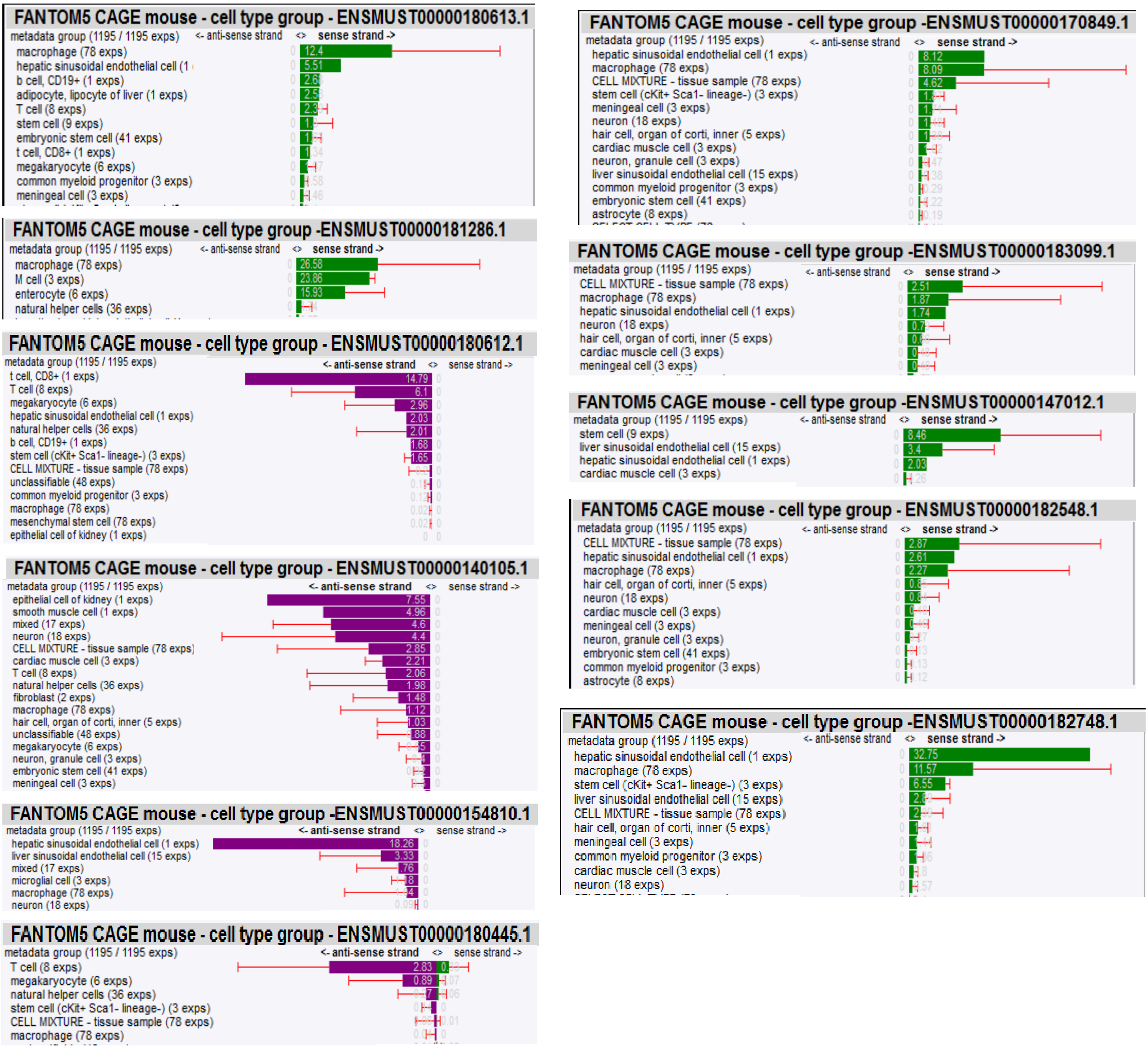

Supplementary Figures S6. Cell-type expression spectrum of the identified lncRNAs. The analysis was carried out using the FANTOM5 Zenbu browser to show which cell types express the identified lncRNAs. Expression level is shown by Tags per million, TPM. Color of expression histogram (green or purple) means that the lncRNAs are coded on genome either forward or reverse direction.

Supplementary Figure S7

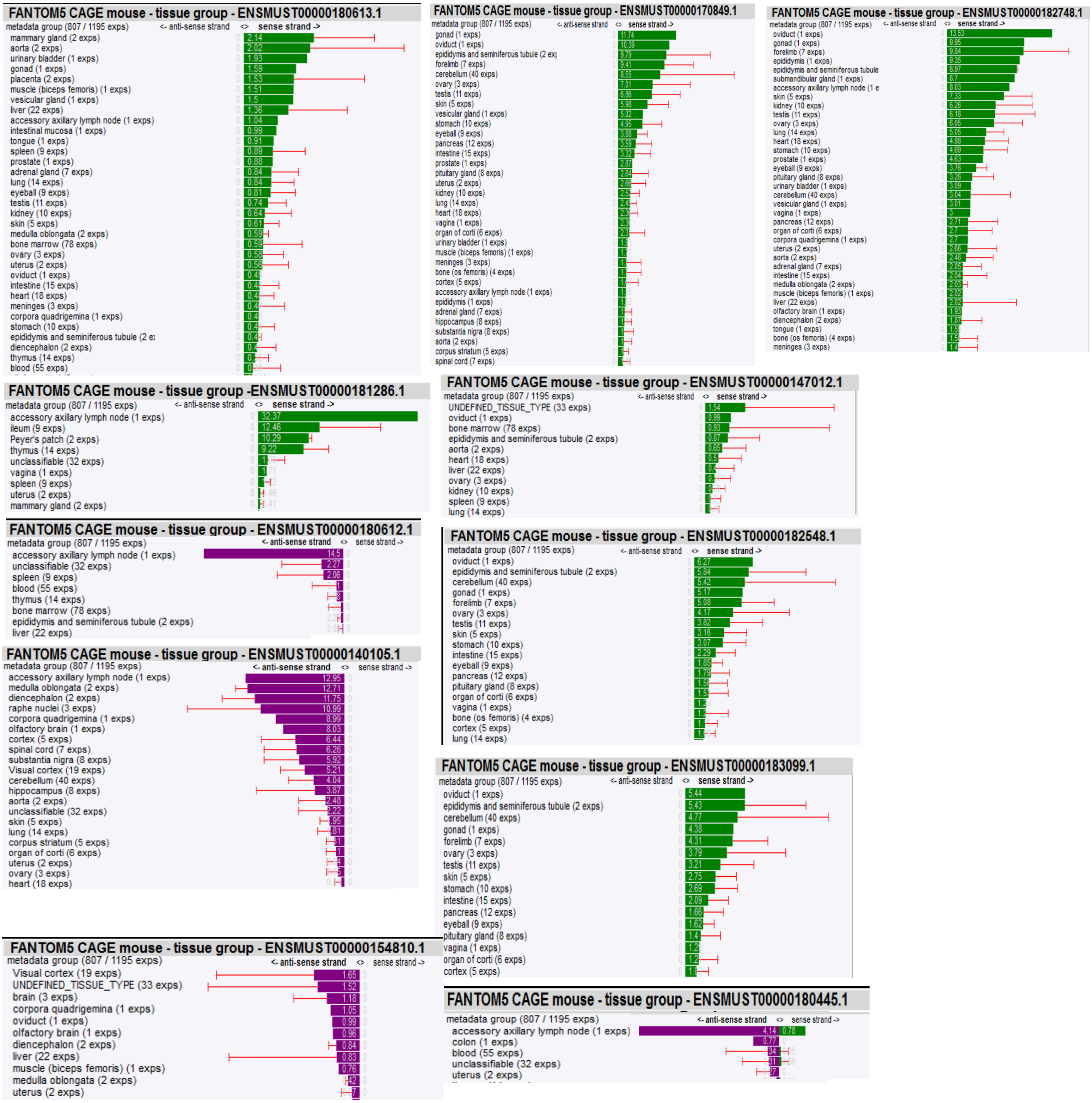

Supplementary Figures S7. Tissue-type expression spectrum of the identified lncRNAs. The analysis was carried out using the FANTOM5 Zenbu browser to show which tissue types express the identified lncRNAs. Expression level is shown by Tags per million, TPM. Color of expression histogram (green or purple) means that the lncRNAs are coded on genome either forward or reverse direction.

Supplementary Figure S8

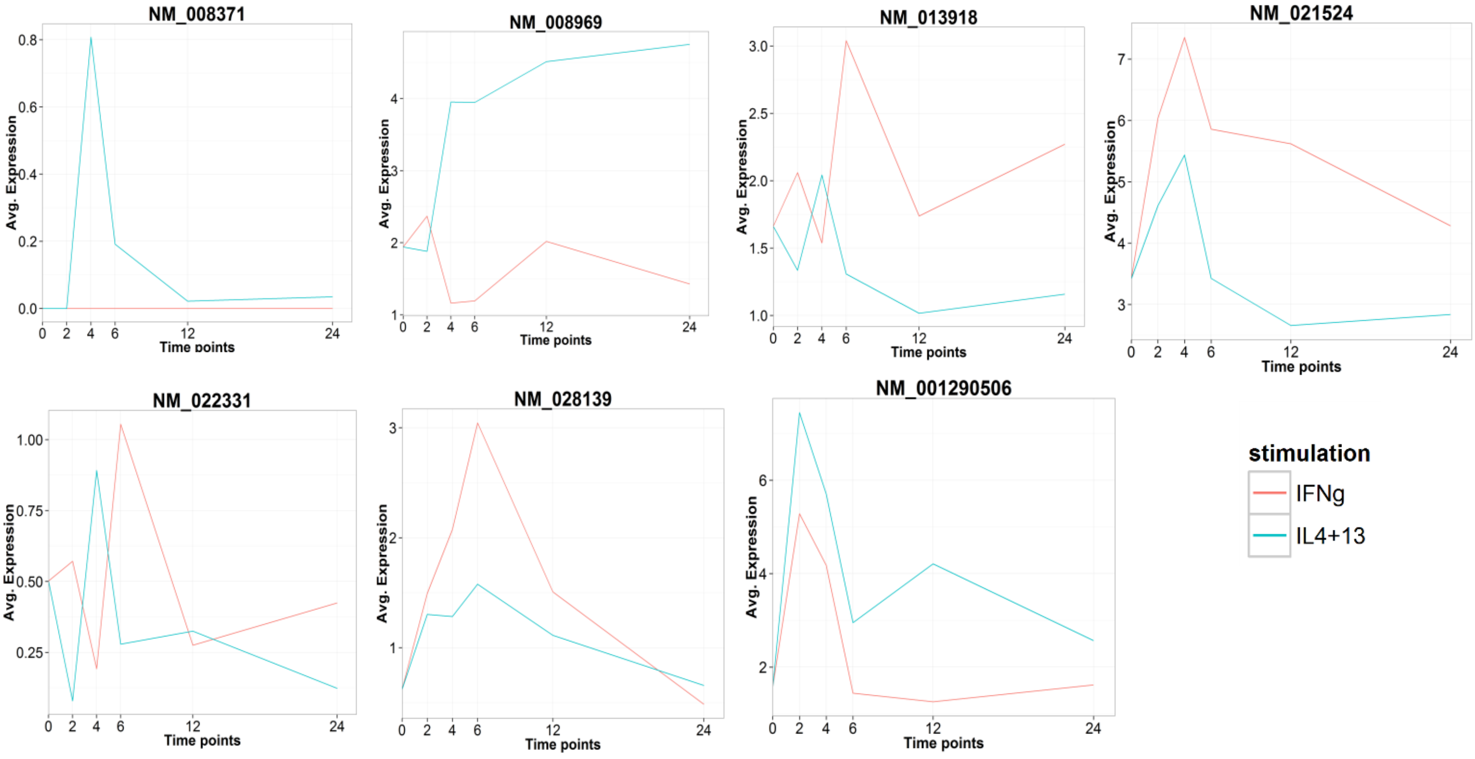

Supplementary Figures S8. Expression Profile of nearby protein coding genes for differentially expressed lncRNA genes. We consider all protein coding genes from Refseq database and find the nearest genes using bedtools with closest option. In most cases, there is no overlap between differentially expressed lncRNA genes and corresponding protein coding genes. The expression profile of protein coding gene in IFNγ-stimulated (in red line) and in IL-4/IL-13-stimulated (in blue line) macrophages was shown. Information of lncRNA genes and their nearest protein coding genes is indicated in the supplementary Table S6.

Supplementary Table legends

Supplementary Table 1. Sample quality assessment. (A) Number of mapped tags on genome in each CAGE library and RIN scores of RNA samples used for the CAGE library preparation are shown. **(B) Differentially expressed up- and down- regulated promoters in unstimulated_24h BMDMs.** Unstimulated_24h BMDMs were compared with unstimulated_0h using edge R to obtain differentially expressed promoters. Promoters, with more than 2-fold change and significant (FDR<0.05) were listed up. Fold changed are represented by logFC and Expression level was shown by Tags per million (TPM).

Supplementary Table 2. Motif Activity Response Analysis of (A) M(IFNγ) and (B) M(IL-4/IL13). Motif activity response analysis was done using promoter activity profile of M(IFNγ) and M(IL-4/IL13) from CAGE data. Motif activity and z-score values were shown in the table.

Supplementary Table 3. Differentially expressed up- and down-regulated TF genes in (A) M(IFNγ) and (B) M(IL-4/IL-13). IFNγ- and IL-4/IL-13-stimulated BMDMs at 2, 4, 6, 12, 24 hours were compared with non-stimulated BMDMs at 0 hours using edge R to obtain differentially expressed transcription factors. Transcription factor, with more than 2-fold change significant (FDR <0.05) at each time point, were listed up. Fold change are represented by logFC values in which significant values are shown in red. Expression level was also shown by Tags per million (TPM).

Supplementary Table 4. Differentially expressed up- and down-regulated non-TF protein coding genes in (A) M(IFNγ) and (B) M(IL-4/IL-13). Differentially expressed protein coding genes, other than transcription factors at 2, 4, 6, 12, 24 hours in IFNγ- and IL-4/IL-13-stimulated BMDMs, were obtained by comparing with non-stimulated BMDMs at 0 hours using edge R. Up- and down-regulated protein coding genes was selected using threshold Log FC >2 and 0.05 FDR. Expression values in Tags per million (TPM) of significantly up- and down-regulated genes are shown in the table in which significant values are shown in red. Newly identified markers in this study are indicated in the table column. PMID (PubMed unique identifier) for previously identified maker genes was also indicated.

Supplementary Table 6. Differentially expressed lncRNA promoters in M(IFNγ) and M(IL-4/IL-13). Differentially expressed lncRNA promoters, at 2, 4, 6, 12, 24 hours in IFNγ- and IL4/IL13-stimulated BMDMs, were obtained by comparing with non-stimulated BMDMs at 0 hour using edge R. Up-regulated lncRNA promoters were selected using threshold Log FC >1 and 0.05 FDR. Expression values in Tags per million (TPM) of significantly up-regulated lncRNA promoters are shown in the table in which significant values are shown in red. Last two column shows list of the nearby protein-coding genes to the corresponding lncRNA gens and their relative distance. Distance zero means they overlap. Negative or positive value of distance means that nearest protein-coding gene resides in up or down stream of corresponding lncRNA gene.

Supplementary table 5A. Gene ontology analysis of commonly induced genes in both IFNγ- and lipid A-stimulation

Rank	GO ontology term	FDR
1	(GO:0006955)immune response	4.245E-14
2	(GO:0006952)defense response	6.57E-09
3	(GO:0006954)inflammatory response	6.78E-09
4	(GO:0009611)response to wounding	2.134E-06
5	(GO:0002694)regulation of leukocyte activation	0.0014714
6	(GO:0050865)regulation of cell activation	0.0016225
7	(GO:0001817)regulation of cytokine production	0.0090571

Supplementary table 5B. Gene ontology analysis of IFNγ-specifically induced genes compared with lipid A

Rank	GO ontology term	FDR
1	(GO:0048002)antigen processing and presentation of peptide antigen	2.256E-05
2	(GO:0002495)antigen processing and presentation of peptide antigen via MHC class II	6.324E-05
3	(GO:0019886)antigen processing and presentation of exogenous peptide antigen via MHC class II	6.324E-05
4	(GO:0002504)antigen processing and presentation of peptide or polysaccharide antigen via MHC class II	0.000134
5	(GO:0002478)antigen processing and presentation of exogenous peptide antigen	0.0003042
6	(GO:0019884)antigen processing and presentation of exogenous antigen	0.0006975
7	(GO:0019882)antigen processing and presentation	0.002356
